# Supplementary material for: Spike sorting with Gaussian mixture models
Source: Sci Rep. 2019 Mar 6;9:3627. doi: 10.1038/s41598-019-39986-6 (PMC6403234; doi:10.1038/s41598-019-39986-6)
Supplement: Supplementary file 1 — Supplementary Material [file 41598_2019_39986_MOESM1_ESM.pdf]

Spike sorting with Gaussian mixture models

Bryan C. Souza, Vítor Lopes-dos-Santos, João Bacelo and Adriano B. L. Tort
Brain Institute, Federal University of Rio Grande do Norte, Natal, Brazil

Supporting Information

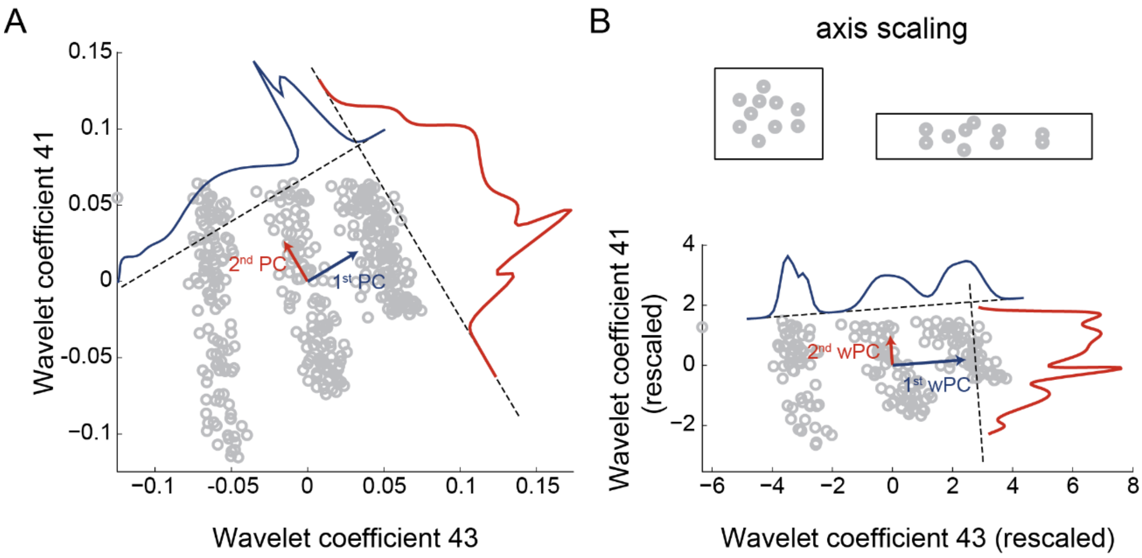

Supplementary Figure S1. Rescaling wavelet coefficients by separability metric. A. Scatter plot of two wavelet coefficients. The first and second principal components (blue and red arrows) are shown along with a Gaussian fit of the data projected onto these axes. **B.** (Top) Scheme of axis rescaling. In the weighted-PCA, each dimension is z-scored and scaled by a particular metric before the computation of the principal components (i.e., the variance of each dimension is weighted). (Bottom) The same as in A after weighting the coefficients by the separability metric. Note that the first weighted principal component (blue curve in B) can better separate the three clusters than the first principal component (blue curve in A).

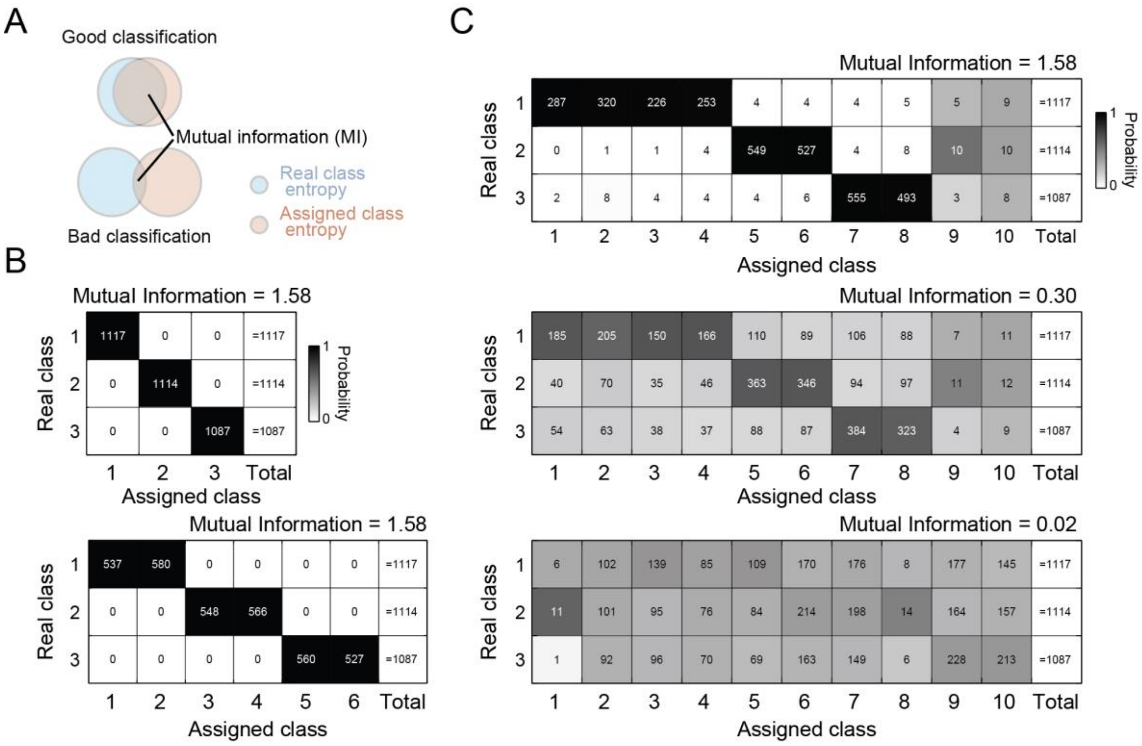

Supplementary Figure S2. Measuring classification performance with Mutual Information (MI). A. Information diagram of real and assigned classes (in our case, neuronal clusters). Good performance implies higher mutual information (MI) between the two groups. **B.** Contingency tables showing perfect classification without (top) and with (bottom) overclustering. Table color denotes the probability (over columns) of each real class to be classified in that particular cluster (assigned class). **C.** Contingency table and MI values for 3 sorting examples differing in classification performance. Note that although the MI values are the same for both cases in B, they are sensitive to the mixing of classes in C. The MI_{norm} shown in the main figures is defined as the normalization of the measured MI value by the maximal possible MI.

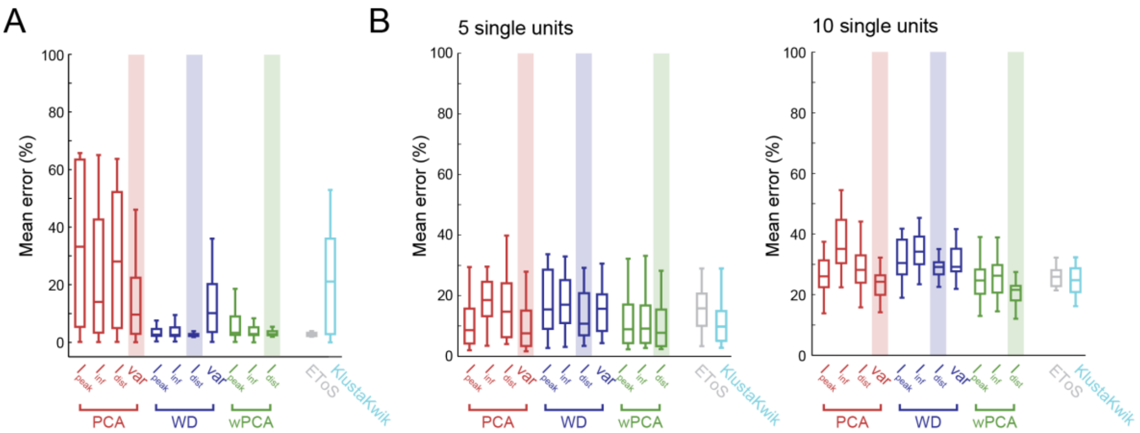

Supplementary Figure S3. Assessing spike sorting performance through mean error. A-B. Boxplots of mean percentage error for each GMM-based feature extraction strategy, EToS and KlustaKwik for (A) Dataset A and (B) Dataset B. Note that the error rates of each method were consistent with their MI_{norm} values (Figs 4 and 5).

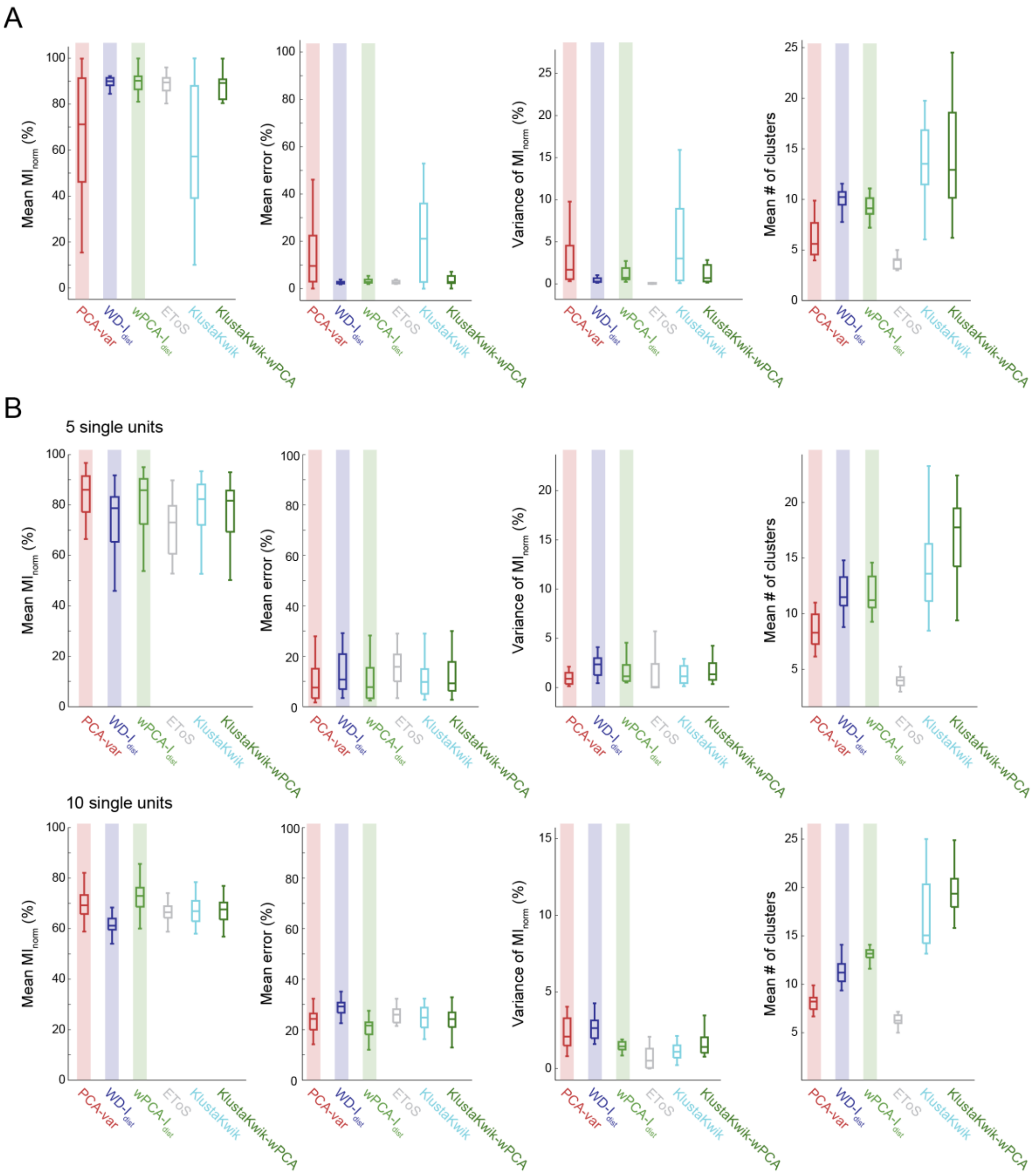

Supplementary Figure S4. Using wPCA-I$_{dist}$ with KlustaKwik. A. Boxplots showing the mean spike information (left), mean error (middle left), consistency (middle right) and mean cluster number (right) for a modified KlustaKwik that used wPCA-I$_{dist}$ instead of PCA for Dataset A. For comparison, the boxplots of EToS, KlustaKwik and each of the best GMM-based methods are also shown. **B.** The same as in A, for Dataset B. Note the increase in performance of KlustaKwik-wPCA in comparison to standard KlustaKwik for Dataset A.

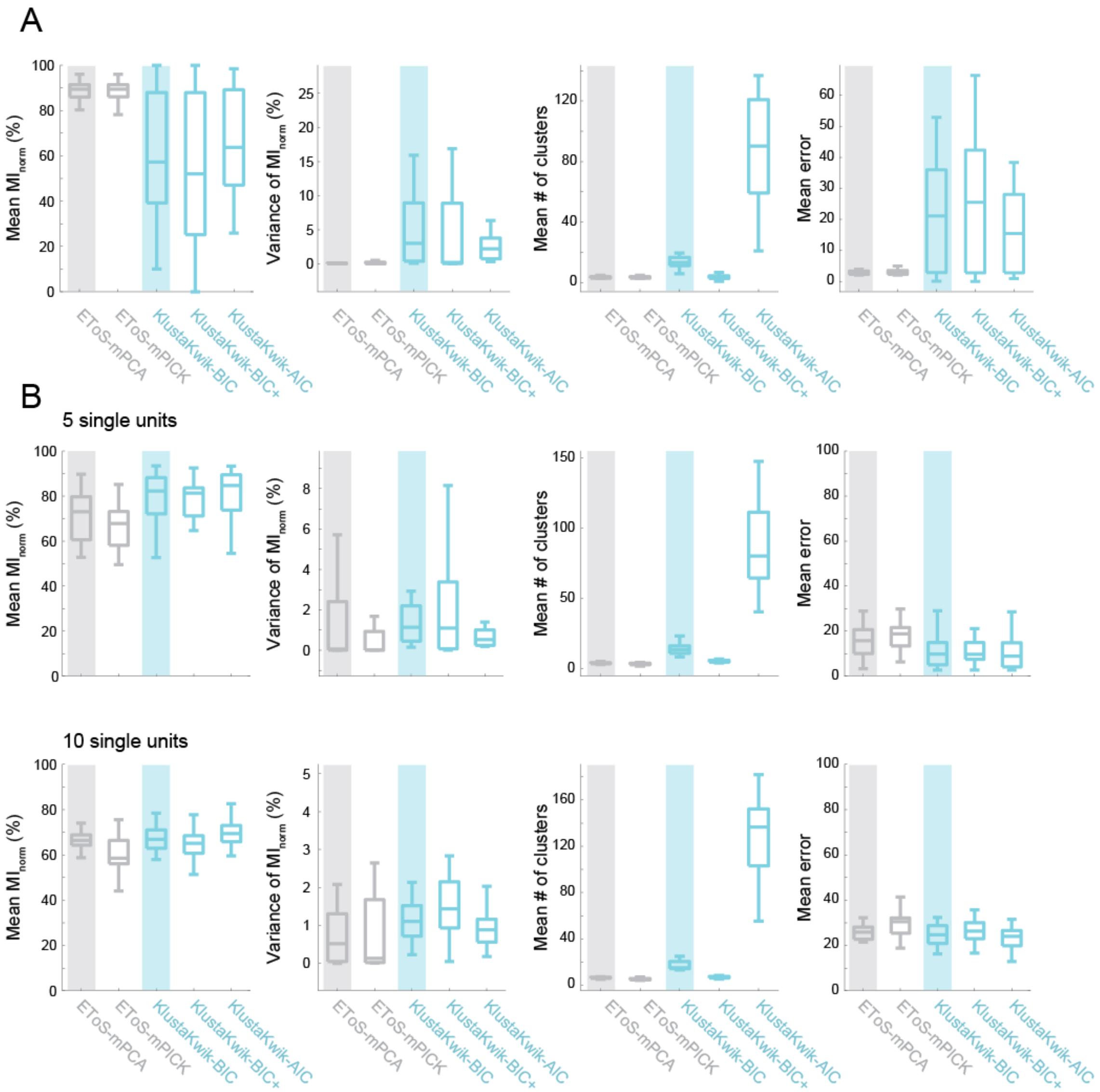

Supplementary Figure S5. Sorting performance under parameter optimization for EToS and KlustaKwik. A. Mean and variance of MInorm, mean number of clusters and mean error of different settings of EToS and KlustaKwik for classification of Dataset A. For EToS, the parameter consisted in a metric of feature selection (i.e., mPCA or mPICK). For KlustaKwik, two parameters defined the penalty of splitting a cluster in two: PenaltyK and PenaltyKLogN. Their values were set, respectively, to (0;1), corresponding to a Bayesian information criterion (BIC), (1;0), corresponding to Akaike's information criterion (AIC) and (0;5), denoted as BIC+. Highlights indicate the original parameters used in the rest of the paper. **B.** Same as in A for Dataset B. Note that KlustaKwik-AIC and KlustaKwik-BIC+ respectively increased and decreased overclustering with no significant changes in performance. Also, EtoS-mPICK showed similar or worse classification performance compared to EtoS-mPCA with no major changes in the number of clusters.

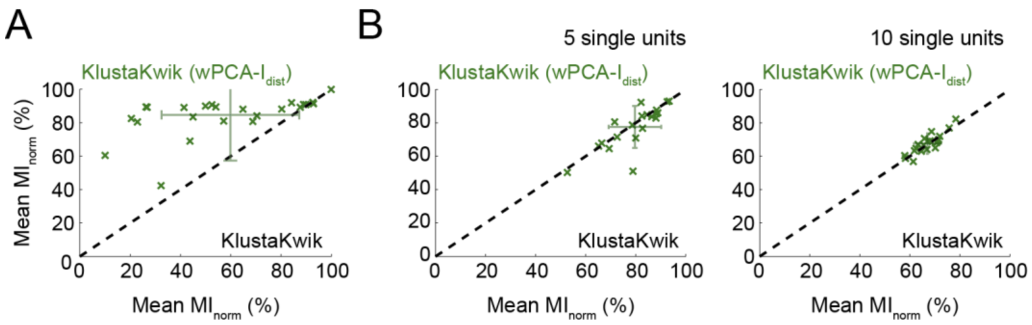

Supplementary Figure S6. Comparing sorting performance of KlustaKwik. A-B. Pairwise comparison between the wPCA-I_{dist} and the standard KlustaKwik for (A) Dataset A and (B) Dataset B. The performance of KlustaKwik using features extracted by wPCA-I_{dist} increased the in comparison to the standard KlustaKwik for Dataset A and remained roughly the same for Dataset B. Errorbars denote standard deviation around the mean.

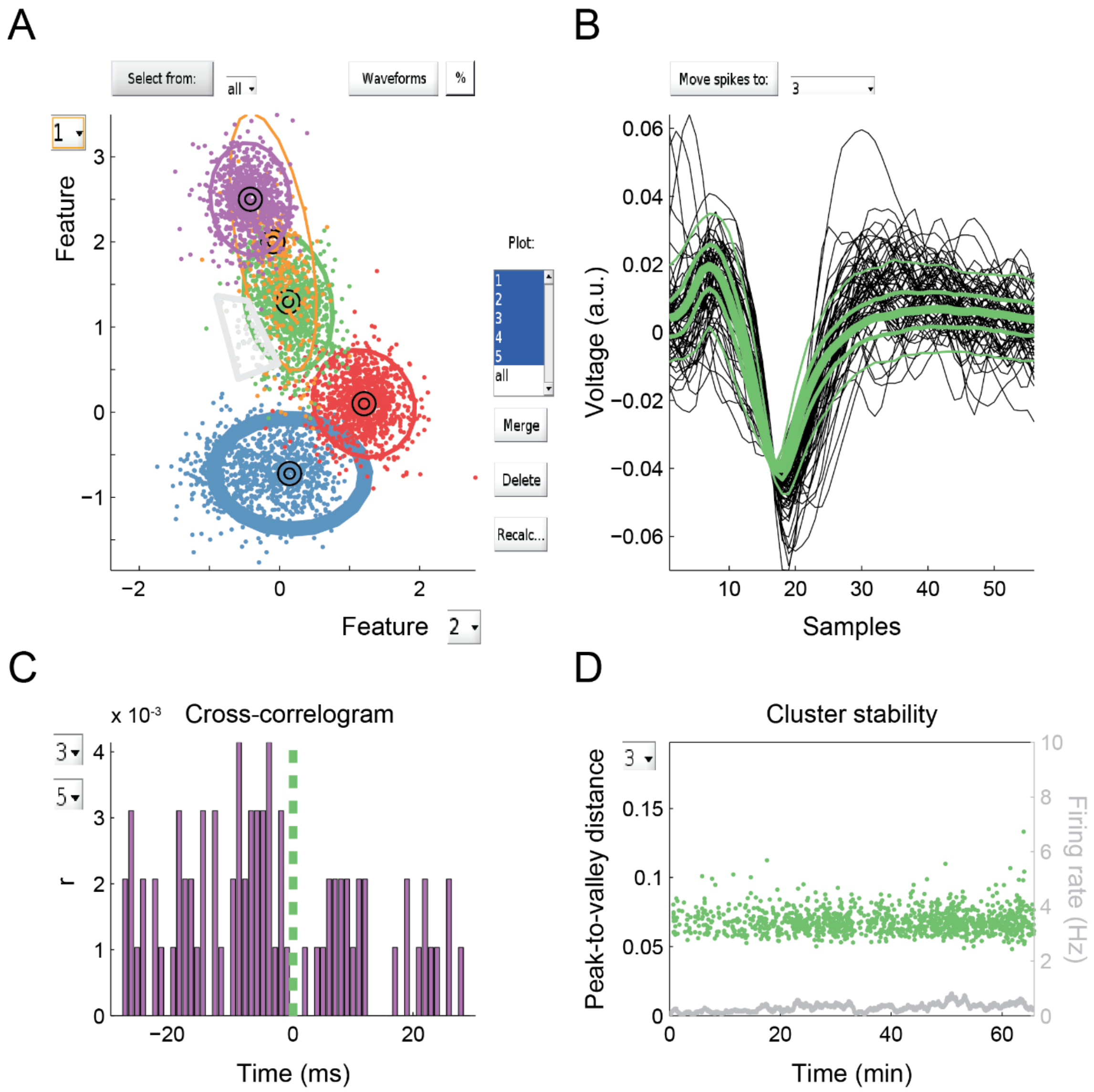

Supplementary Figure S7. Example of a screen of the developed GUI. A. Feature space showing the first two out of five features. Ellipses demark Gaussians defining clusters. Gray polygon was selected manually for further inspection in B. **B.** Mean waveform for cluster 3 (green; thin lines show quantiles). Black waveforms correspond to the gray dots selected in A. **C.** Cross-correlogram between clusters 3 and 5. **D.** Peak-to-valley distance for individual waveforms and firing rate showing the stability of cluster 3 along the recording time. MATLAB scripts and GUI instructions are available at https://github.com/tortlab/GMM-spike-sorting.

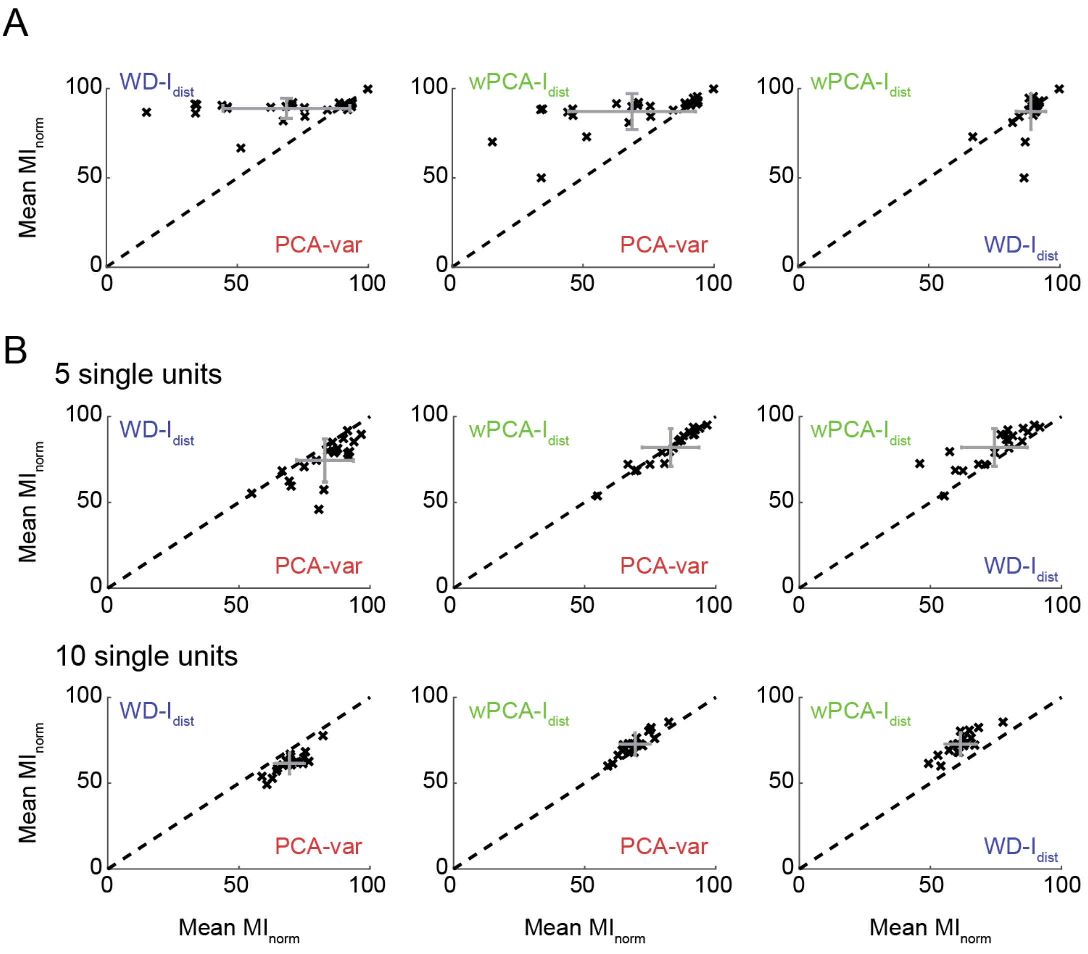

Supplementary Figure S8. Comparing sorting performance between best strategies. A-B. Pairwise comparison between the WD-I_{dist}, wPCA-I_{dist} and PCA-var for (A) Dataset A and (B) Dataset B. The performance of wPCA-I_{dist} was either similar to or better than any of the other approaches. Errorbars denote standard deviation around the mean.

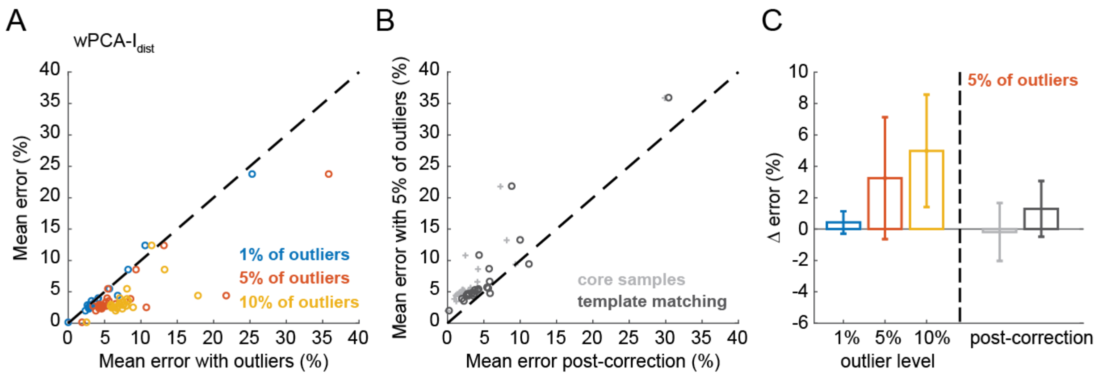

Supplementary Figure S9. Sorting performance with outliers. A. Pairwise comparison between the mean error of wPCA-I_{dist} under different outlier levels (Dataset A). Outliers were created by adding waveforms, simulating simultaneous spikes. **B.** Comparison of the mean error found with 5% of outliers and two post-processing steps (see Discussion). **C.** The left colored bars show difference in mean error due to outliers (m ± s.d.). The right bars show the difference in mean error for 5% percent of outliers under the two correction approaches. Note that the error increases with the number of outliers but can be reversed to the original levels when considering only the core samples, defined as 95% of the samples with smallest k-nearest neighborhood distance (k=10). Reclassifying the non-core samples using a template matching approach helps to reduce the error introduced by the outliers.

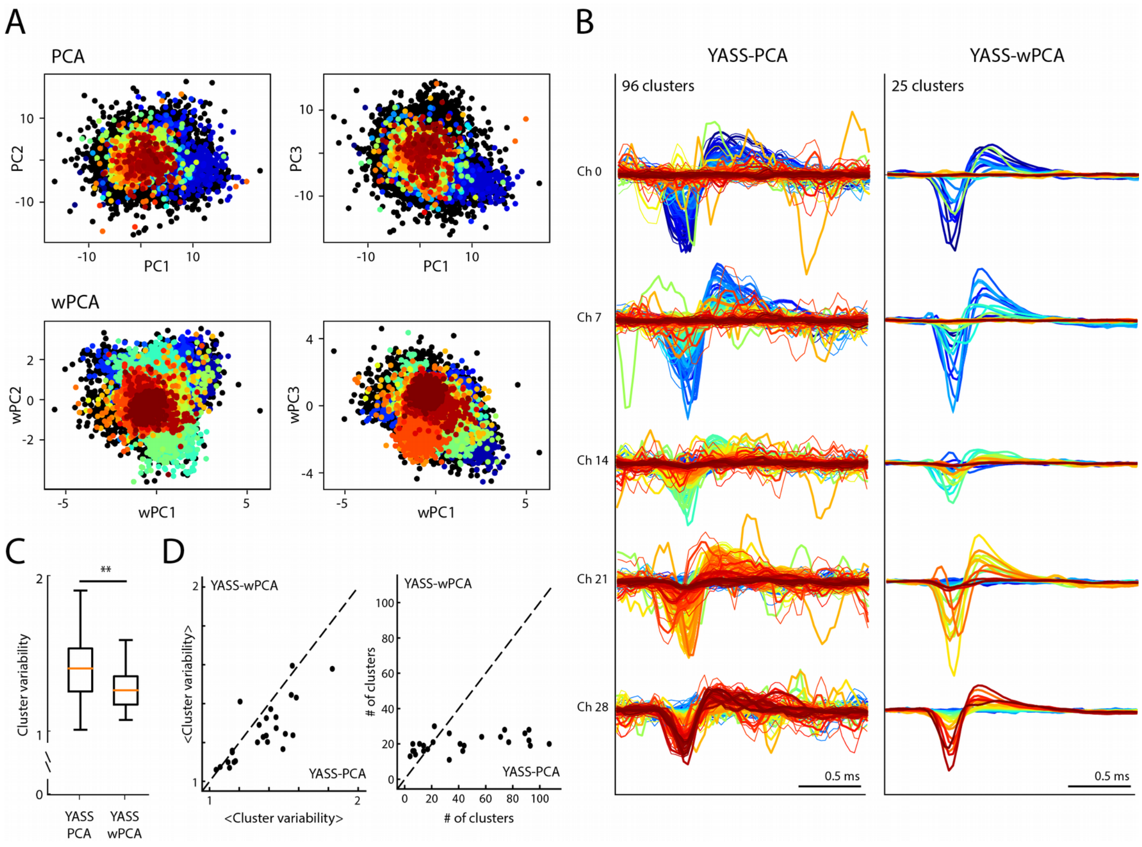

Supplementary Figure S10. Comparing PCA and wPCA strategies to unsupervised spike sorting with YASS. A. Recordings from a Neuropixel probe (data from https://doi.org/10.6084/m9.figshare.7666892.v1) were used for testing YASS performance with PCA (YASS-PCA) or wPCA-I_{dist} (YASS-wPCA). Panels show the first three principal components extracted from an example dataset using PCA (top) and wPCA (bottom). Black and colored dots represent unclassified and classified waveforms, respectively. **B.** Mean cluster waveforms in five example channels are shown for YASS-PCA (left; 96 clusters found) and YASS-wPCA (right; 25 clusters found). Colors code for the same clusters as in A. Notice that YASS-wPCA yields well-defined waveforms that can be better discriminated in the feature space. **C.** Distribution of waveform variability within each cluster for an example dataset. Waveform variability was computed as the average of the standard deviation of each time point. **D.** Group data (n=25 datasets). Note that waveform variability is lower for the YASS-wPCA even though it finds a lower number of clusters. In these analyses, each dataset was composed of 30 adjacent channels out of 385.

Supplementary Table S1. List of datasets used.

	File	# of detected spikes
Dataset A (Quiroga et al., 2004)	C_Difficult1_noise005.mat	3182
	C_Difficult1_noise01.mat	3318
	C_Difficult1_noise015.mat	3351
	C_Difficult1_noise02.mat	3293
	C_Difficult2_noise005.mat	3200
	C_Difficult2_noise01.mat	3360
	C_Difficult2_noise015.mat	3319
	C_Difficult2_noise02.mat	3286
	C_Easy1_noise005.mat	3343
	C_Easy1_noise01.mat	3376
	C_Easy1_noise015.mat	3327
	C_Easy1_noise02.mat	3313
	C_Easy1_noise025.mat	2802
	C_Easy1_noise03.mat	2217
	C_Easy1_noise035.mat	1486
	C_Easy1_noise04.mat	983
	C_Easy2_noise005.mat	3258
	C_Easy2_noise01.mat	3379
	C_Easy2_noise015.mat	3252
	C_Easy2_noise02.mat	3288
	C_Burst_Easy2_noise015	3259
	C_Drift_Easy2_noise015	2993
	C_Easy1_noise01_short	521
	C_Test_LFPcorr_Easy2_noise015	3184
	times_C_Difficult1_noise015	3184
Dataset B (CRCNS: hc1)	Comb5 1 (d14521.001, d1453101, d1512103, d561104, d561105)	1709
	Comb5 2 (d11222.002, d14521.001, d533101, d561104, d561106)	6631
	Comb5 3 (d11222.002, d1453103, d1453104, d533101, d561105)	9680
	Comb5 4 (d11222.002, d12821.001, d1453104, d561104, d561105)	9880
	Comb5 5 (d11222.001, d12821.001, d1453101, d1453104, d533101)	7133
	Comb5 6 (d1282101, d1453103, d1512103, d561103, d561105)	2838
	Comb5 7 (d1282101, d533101, d561104, d561105, d561106)	2878
	Comb5 8 (d11222.001, d12821.001, d1453103, d533101, d561105)	5464
	Comb5 9 (d1282101, d1512103, d561103, d561104, d561106)	2554
	Comb5 10 (d11222.002, d1282101, d1512103, d533101, d561106)	6245
	Comb5 11 (d12821.001, d1282101, d1453103, d561103, d561106)	4820
	Comb5 12 (d1282101, d1453101, d1453103, d561105, d561106)	3127
	Comb5 13 (d12821.001, d14521.001, d561103, d561104, d561105)	3440
	Comb5 14 (d1512103, d533101, d561104, d561105, d561106)	2537
	Comb5 15 (d11222.002, d12821.001, d1282101, d533101, d561105)	7431
	Comb5 16 (d11222.002, d12821.001, d1453101, d561103, d561104)	7721
	Comb5 17 (d11222.002, d12821.001, d1453103, d1453104, d561103)	10940
	Comb5 18 (d11222.002, d14521.001, d533101, d561103, d561106)	6361
	Comb5 19 (d1282101, d1453103, d1453104, d1512103, d561104)	5876
	Comb5 20 (d11222.002, d1453101, d561103, d561104, d561106)	6573
	Comb10 1 (d11222.002, d12821.001, d14521.001, d1453101, d1453103, d1453104, d1512103, d533101, d561103, d561105)	12665
	Comb10 2 (d1282101, d14521.001, d1453101, d1453103, d1453104, d1512103, d533101, d561104, d561105, d561106)	8037
	Comb10 3 (d1282101, d14521.001, d1453101, d1453104, d1512103, d533101, d561103, d561104, d561105, d561106)	7085
	Comb10 4 (d11222.001, d12821.001, d1282101, d14521.001, d1453104, d1512103, d533101, d561103, d561104, d561106)	9459
	Comb10 5 (d11222.001, d11222.002, d1282101, d14521.001, d1453101, d1453103, d1453104, d533101, d561103, d561106)	12917
	Comb10 6 (d11222.001, d1282101, d14521.001, d1453101, d1453103, d1453104, d533101, d561104, d561105, d561106)	9134
	Comb10 7 (d11222.001, d11222.002, d12821.001, d1282101, d14521.001, d1453103, d1453104, d1512103, d533101, d561105)	13418
	Comb10 8 (d11222.001, d1282101, d1453101, d1453103, d1453104, d1512103, d533101, d561104, d561105, d561106)	9093
	Comb10 9 (d11222.001, d11222.002, d12821.001, d1282101, d14521.001, d1453101, d1453103, d1453104, d533101, d561105)	13687
	Comb10 10 (d11222.002, d12821.001, d1282101, d1453101, d1453103, d1453104, d533101, d561103, d561104, d561106)	14049
	Comb10 11 (d11222.001, d11222.002, d1282101, d14521.001, d1453103, d1453104, d1512103, d533101, d561105, d561106)	12270
	Comb10 12 (d11222.001, d11222.002, d12821.001, d1282101, d14521.001, d1453103, d1512103, d561103, d561105, d561106)	10762
	Comb10 13 (d11222.001, d11222.002, d12821.001, d14521.001, d1453101, d1453103, d1453104, d561104, d561105, d561106)	13766
	Comb10 14 (d11222.002, d1282101, d14521.001, d1453101, d1453103, d1453104, d1512103, d561104, d561105, d561106)	11426
	Comb10 15 (d11222.001, d11222.002, d14521.001, d1453101, d1453104, d1512103, d533101, d561104, d561105, d561106)	11516
	Comb10 16 (d11222.001, d11222.002, d12821.001, d1282101, d1453101, d1453104, d533101, d561104, d561105, d561106)	13400
	Comb10 17 (d11222.001, d11222.002, d1282101, d14521.001, d1512103, d533101, d561103, d561104, d561105, d561106)	9198
	Comb10 18 (d11222.001, d12821.001, d1282101, d14521.001, d1453101, d1453103, d1453104, d561103, d561105, d561106)	9746
	Comb10 19 (d11222.001, d11222.002, d1282101, d1453101, d1453104, d1512103, d533101, d561103, d561104, d561106)	12194
	Comb10 20 (d11222.002, d12821.001, d1282101, d14521.001, d1453101, d1453103, d533101, d561103, d561104, d561106)	11284
